# AURORA: A New Dawn

**DOI:** 10.1093/cid/ciae114

**Published:** 2024-04-04

**Authors:** Genovefa A Papanicolaou, Robin K Avery, Catherine Cordonnier, Rafael F Duarte, Shariq Haider, Johan Maertens, Karl S Peggs, Carlos Solano, Jo-Anne H Young, Martha Fournier, Rose Ann Murray, Jingyang Wu, Tien Bo, Drew J Winston

**Affiliations:** Memorial Sloan Kettering Cancer Center, New York, New York, USA; Johns Hopkins University, Baltimore, Maryland, USA; Haematology Department, Henri Mondor Hôpital, Assistance Publique-Hopitaux de Paris, and Université Paris-Est-Créteil, Créteil, France; Department of Haematology, Hospital Universitario Puerta de Hierro Majadahonda, Madrid, Spain; Juravinski Hospital and Cancer Center, Hamilton Health Sciences Corporation, Hamilton, Ontario, Canada; Haematology Department, University Hospitals Leuven, KU Leuven, Leuven, Belgium; Department of Haematology, University College London Hospitals NHS Foundation Trust, London, United Kingdom; Hematology Department, Hospital Clínico Universitario, University of Valencia, Valencia, Spain; University of Minnesota, Minneapolis, Minnesota, USA; Takeda Development Center Americas, Inc, Lexington, Massachusetts, USA; Takeda Development Center Americas, Inc, Lexington, Massachusetts, USA; Takeda Development Center Americas, Inc, Lexington, Massachusetts, USA; Takeda Development Center Americas, Inc, Lexington, Massachusetts, USA; Department of Medicine, University of California, Los Angeles Medical Center, Los Angeles, California, USA

To the  Editor—We appreciate the points raised by Camargo and the opportunity to respond.

To our knowledge, AURORA was the first randomized, double-blind study to compare 2 oral antiviral agents with a primary end point of cytomegalovirus (CMV) clearance. Identified as limitations of AURORA were the 8-week treatment period (rather than stopping treatment upon viral clearance [1]), dose interruption/reduction of valganciclovir in patients with grade 3 neutropenia, and the rate of treatment-emergent resistance. The 8-week treatment period, which may have overexposed patients to valganciclovir vs clinical practice, was necessary to maintain the double-blind study design and benefit from the efficacy and safety of 8 weeks of maribavir treatment [2, 3]. The primary end point was viremia clearance at week 8, unlike in clinical practice, where the goal is to prevent infection recurrence and end-organ disease by controlling CMV viremia until durable cell-mediated immunity is recovered. Therefore, AURORA required strategies to avoid toxicities and mitigate overexposure/myelosuppression while enabling comparable treatment doses between arms, including dose adjustments, granulocyte colony-stimulating factor use, and therapy interruption [4]. In addition, an original inclusion criterion for AURORA of a viral load (VL) >910 IU/mL was later amended to allow entry of high-risk patients with a VL >455 IU/mL, in line with clinical practice [4, 5]. These patients represented 18% of the study population [4]. Stringent hematology parameters were also required for study entry, likely resulting in underestimation of valganciclovir discontinuation compared with clinical practice. Last, treatment-emergent resistance to maribavir occurred in 9% of patients in AURORA [4], whereas the 26% rate noted was for the refractory CMV patient population in SOLSTICE [6]. Most maribavir breakthrough infections in AURORA were in those with maribavir resistance and cleared after switching to treatments with different mechanisms of action 4].

Acknowledging these limitations, AURORA provided clinically relevant, actionable data [4]. Comparable viremia clearance was achieved during 8 weeks of treatment (maribavir: 83%; valganciclovir: 86%) [4]. Patients had fewer treatment-emergent adverse events (TEAEs), hospitalizations, and TEAE-related discontinuations with maribavir than with valganciclovir, with no maribavir-related deaths [4]. More recurrent CMV infections occurred after the 8-week treatment period with valganciclovir (23% vs 19%) [4]. CMV recurrence has been reported in up to 50%–70% of patients after preemptive therapy [7], likely because maintenance therapy cannot be provided due to the safety and tolerability profiles of conventional agents [8–10]. The authors pose that maintenance therapy at effective doses is a tool to prevent recurrence and associated negative outcomes [11], and this should be confirmed with prospective trials. AURORA confirmed the anti-CMV activity of maribavir with less neutropenia compared with valganciclovir for CMV infection after hematopoietic stem cell transplant [4].

Maribavir's efficacy and safety profile enables clinicians to use it among patients who are at risk of/are experiencing intolerances (eg, myelosuppression, nephrotoxicity) and those who fail to achieve >90% decline in VL after 2 weeks of conventional treatment [4, 12]. These features provide a real benefit for transplant patients despite AURORA not demonstrating noninferiority (within the prespecified 7% margin) of maribavir on CMV viremia clearance at week 8 over valganciclovir. Well-designed, real-world studies are indeed necessary to optimize outcomes with the expanding anti-CMV armamentarium.

## Notes

***Financial support*.** This work was supported by Takeda Development Center Americas, Inc., Lexington, Massachusetts.
